# Assessing the economic burden and health-related quality of life in chinese patients with fibrodysplasia ossificans progressiva: a questionnaire survey analysis

**DOI:** 10.1186/s13023-025-03884-0

**Published:** 2025-08-07

**Authors:** Xiaoyang Xu, Xiaoning He, Wei Wu, Yuhang Xin, Qingnan Li, Lei Song, Kexin Li, Jing Wu

**Affiliations:** 1 School of Pharmaceutical Science and Technology, Faculty of Medicine, Tianjin University, Tianjin, 300072 China; 2 Center for Social Science Survey and Data, Tianjin University, Tianjin, 300072 China; 3 China Alliance for Rare Diseases, Beijing, 100020 China; 4 Medical Price Branch of Beijing Municipal Price Association, Beijing, 100166 China

**Keywords:** Fibrodysplasia ossificans progressive, Economic burden, Health-related quality of life, Real-world

## Abstract

**Background:**

Fibrodysplasia ossificans progressiva (FOP) is an ultra-rare, disabling genetic disorder condition that lacks real-world evidence on disease burden. This study aims to investigate the basic characteristics, diagnostic status, prognosis, economic burden and quality of life of FOP patients in China through self-reported data .

**Methods:**

An online survey was conducted through the “China Cloud Platform for Rare Diseases” among patients recruited from the FOP patient organization in March 2023. Patient demographic characteristics, diagnosis, prognosis, healthcare resource usage and costs, and health-related quality of life (HRQoL) data were collected. Health-related quality of life was assessed using EQ-5D-Y for children aged 4–15 and EQ-5D-5L for patients aged ≥ 16. Age subgroup analyses for those aged < 8, 8–15, and ≥ 16 were conducted.

**Results:**

A total of 67 patients (mean age 16.6 ± 10.2 years, 43.3% female) were included. The average delay of confirmed diagnosis was 3.1 ± 4.3 years. 98.5% of patients were disabled due to FOP. Only 38.8% of the patients had outpatient or inpatient visits in the past year. The annual cost per patient was USD 10,820 ± 10,894, with 75.2% being indirect costs. Health utility values were lowest for patients aged ≥ 16 (0.221 ± 0.336), compared to those aged < 8 (0.700 ± 0.163) and those aged 8–15 (0.618 ± 0.202).

**Conclusion:**

FOP patients suffer long delay diagnosis duration, high disability rates in China. A significant disease burden was driven by high indirect costs and poor quality of life. Patients aged ≥ 16 have the worst health-related quality of life.

**Supplementary Information:**

The online version contains supplementary material available at 10.1186/s13023-025-03884-0.

## Introduction

Fibrodysplasia ossificans progressiva (FOP) is an ultra-rare, disabling genetic condition characterized by congenital malformations of the great toes and progressive heterotopic ossification (HO) in specific anatomic patterns [1]. FOP is caused by a mutation in the activin receptor IA (ACVR1) gene, and approximately 97% of all FOP patients have the classic heterozygous mutation (c.617G > A; p.R206H) [1]. The estimated prevalence of FOP is 1 in 2 million with no predisposition to racial, sex, or geographic distribution. [2]. FOP significantly affects patients'accommodations, mobility, activities of daily living, and social activities, leading to an extremely poor quality of life for the patients, which is called the most catastrophic disorder of heterotopic ossification in humans [1, 3]. Most patients require a wheelchair by their third decade of life [1]. Furthermore, complications such as fractures and severe restrictive lung disease further increase the disease burden of FOP, and the lethal cardiorespiratory failure due to thoracic insufficiency syndrome and pneumonia is the biggest cause of death [4]. The median age of death for FOP patients is only 40 years [4].

FOP is usually diagnosed based on clinical symptoms of skeletal malformations including malformed great toes, soft tissue swelling, and progressive heterotopic ossification, combined with genetic confirmation (ACVR1 gene mutation) [1]. Due to its rarity, clinicians are often unable to associate the classical symptoms with a confirmed FOP diagnosis immediately, resulting in a high misdiagnosis rate, delayed diagnosis and treatment, and unwarranted interventions which cause irreversible harm and further speed the progression of FOP [5]. According to an international survey, the misdiagnosis rate of FOP approached 87% worldwide and the mean period from the onset of symptoms to a confirmed FOP diagnosis was 4.1 years [6]. There is still no proven effective prevention or treatment for FOP [7]. Based on further understanding of the molecular and cellular pathological mechanisms in FOP, newly developed drugs include palovarotene, garetosmab, etc., which may provide breakthrough therapeutic advancements for FOP in the near future [1].

Grasping the empirical data on disease burden for FOP is necessary for medication development, disease management, and resource allocation. Previous empirical research has focused primarily on the nature history and epidemiology of FOP [8–12]. Up to now, insufficient attention has been devoted to the economic burden and quality of life of FOP patients, particularly for a thorough assessment of the costs and reduced quality of life incurred by patients due to FOP [11–14]. Furthermore, the differences in disease burden among different age subgroups are important in progressive diseases, which is however seldomly investigated among patients with FOP in previous research [8, 11, 15].

China, as a developing country, harbors an estimated 20 million rare disease patients. In recent years, the country has demonstrated a dedicated effort towards enhancing diagnostic and treatment standards, along with fortifying the health security system tailored for rare diseases. FOP is one of the 86 diseases of China's second Catalogue of Rare Diseases in 2022 [16]. There were fewer than 150 patients identified by 2018 from the main medical centers of China. However, the absence of comprehensive disease burden data for FOP patients in China significantly impedes disease management, future drug reimbursement access, etc. This study aims to comprehensively explore the disease burden among Chinese FOP patients via a self-reported questionnaire and conduct analyses among distinct age subgroups.

## Methods

### Overview

An online survey was administered in March 2023 through the “China Cloud Platform for Rare Disease” in China. Participants in this study were recruited through the Chinese FOP patient organization “The Angel's Coral”, which comprises approximately 80 patients and is currently the biggest FOP patient association in China. The information regarding the patient's demographic characteristics, diagnosis and prognosis, healthcare resource usage and costs, and health-related quality of life were collected and analyzed. FOP patients’ basic characteristics, diagnosis and prognosis status, and disease burden were quantified for the total population and the age-specific subgroups (< 8 years, 8–15 years, and ≥ 16 years).

### Questionnaire design and data collection

The structured questionnaire was designed by researchers from Tianjin University and China Alliance for Rare Diseases based on a literature review, and refined based on feedback received from Chinese clinical experts to ensure a more accurate depiction of the symptoms and medical experience of FOP patients. Before the formal investigation, a pilot survey was conducted on 3 patients (each from one age subgroup) first, and further refinements in the questionnaire were made accordingly.

All of the patients with FOP were eligible for the survey, and quota for age subgroups including < 8 years, 8–15 years, and ≥ 16 years were set to ensure the representativeness of the study sample. Patients or their guardians were first contacted through the WeChat. Participants who agreed to participate were required to sign an informed consent and then answer the survey online. The questionnaire can be independently completed by the patients, either with or without assistance from their guardians, or it can be completed by their guardians on behalf of the patients. Data quality control was conducted after the survey. Individual telephone follow-ups were performed for questionnaires exhibiting logical errors, anomalous data, or missing information, and data were corrected accordingly. Ethical approval of this study was obtained from the Ethics Committee (blinded for review).

## Measures

### Patient basic characteristics

Patient basic characteristics included sociodemographic information and family history of disease. Sociodemographic information consisted of sex, age, BMI, insurance coverage category, highest educational level, etc.

### Diagnosis and prognosis

The patient's diagnostic journey was comprehensively outlined, including the age of onset, delayed diagnosis duration, misdiagnoses, iatrogenic harms and type of FOP, etc. Prognostic indicators included the progression of HO, burden of associated complications, and disability. The disability was assessed through self-reported disability, information of disability certification (type, grade), use of assistive devices, etc. In China, disability certifications classify disabilities into seven types which are physical, hearing, vision, speech, mental, intellectual, and multiple disabilities, with grades range from severe to mild on a scale of 1 to 4.

### Economic burden

Economic burden was assessed from the societal perspective including per capita healthcare resource utilization and costs. Healthcare resource utilization encompassed the annual number of outpatient and hospitalisation visits. The annual per capita costs comprised direct medical costs, direct non-medical costs, and indirect costs. Direct medical costs included outpatient and hospitalisation costs, while direct non-medical costs encompassed transportation, accommodation, nursing, nutrition, assistive devices, and other related costs. Indirect costs covered the patient's and their relatives'unemployment and absenteeism loss due to FOP. The patients’ wage was calculated using the 2023 China Gross Domestic Product (GDP) per capita value of ¥89,358. The annual per capita healthcare resource utilization and costs were calculated by multiplying the healthcare resource utilization and costs reported in the questionnaire for the past 3 months by a factor of 4.

The total direct medical costs from the disease onset to the time of the survey were also considered including direct medical costs incurred before diagnosis due to misdiagnosis and costs from the time of diagnosis to the survey. All costs were presented in USD ($). Cost data were converted using the 2023 annual average exchange rate of Chinese Yuan Renminbi (CNY) to US Dollar (USD) (1 USD = 7.08 CNY), sourced from the International Monetary Fund (IMF, 2023).

### Health-related quality of life

Health-related quality of life was assessed using the EuroQol Five-Dimensional Questionnaire, Youth Version (EQ-5D-Y) or the EuroQol Five-Dimensional Questionnaire, Five-Level Version (EQ-5D-5L) for children aged 4–15 and adult patients aged ≥ 16, respectively. Both instruments comprise five dimensions including mobility, self-care, usual activities, pain/discomfort, and anxiety/depression. The EQ-5D-Y has 3 levels per dimension, while the EQ-5D-5L has 5 levels per dimension. Higher levels indicate more severe issues within each dimension. Patients'health-related quality of life was expressed in utility values calculated using the Chinese health utility value set, where higher utility values reflect better life quality [17, 18].

## Statistical analyses

Descriptive analyses of basic characteristics, diagnosis and prognosis, economic burden and quality of life were carried out for the total population and subgroups. Summaries of continuous variables were reported with arithmetic mean, standard deviation (SD), median, minimum, maximum and 95% confidence interval. Categorical data were summarized using frequencies and percentages. All statistical analyses in this study were performed using STATA 16.0 software.

## Results

### Basic characteristics

A total of 67 patients were included in March 2023 across three subgroups of patients aged < 8 (N = 17), 8–15 years (N = 18), and ≥ 16 years (N = 32). In the overall population, patient-reported outcomes indicated that the average age was 16.6 ± 10.2 years, and 43.3% were female. 94% of the patients were covered by basic medical insurance. Around 2/3 (62.7%) of patients were absent from school or unemployed due to FOP. More than half of the patients (53.0%) annual household income was below ¥50,000. None of these patients had a family history of the disease. **(**Table 1**).**

**Table 1:** Basic characteristics of FOP patients

Variables	Total population N = 67	Age 0–8 N = 18	Age 8–15 N = 17	Age ≥ 16 N = 32
*Sociodemographic characteristics*				
Females, n (%)	29 (43.3%)	5 (27.8%)	9 (52.9%)	15 (46.9%)
Age, years, mean (SD)	16.6 (10.2)	5.7 (1.2)	11.5 (1.9)	25.5 (7.4)
95% Confidence intervals	(14.2,19.1)	(5.1,6.4)	(10.5,12.5)	(22.8,28.2)
Median (range)	13.8 (3.4,48.7)	6.1 (3.4,7.7)	11.8 (8.4,14.9)	25.5 (16.1,48.7)
BMI, kg/m^2^, mean (SD)	18.4 (4.7)	16.6 (3.6)	18.9 (5.6)	19.1 (4.5)
95% Confidence intervals	(17.2,19.5)	(14.8,18.4)	(16.0,21.8)	(17.5,20.7)
Median (range)	17.4 (11.0,32.0)	15.5 (11.8,24.9)	16.8 (12.4,31.9)	18.0 (11.0,29.0)
Insurance coverage, n (%)	63 (94.0%)	18 (100.0%)	17 (100.0%)	28 (87.5%)
Basic medical insurance^1^	63 (94.0%)	18 (100.0%)	17 (100.0%)	28 (87.5%)
Other insurance^2^	6 (9.0%)	3 (16.7%)	3 (17.6%)	4 (12.5%)
None	4 (6.0%)	0 (0.0%)	0 (0.0%)	4 (12.5%)
*Highest educational level, n (%)*				
Elementary school and below	41 (61.2%)	18 (100.0%)	13 (76.5%)	10 (31.3%)
Middle school	14 (20.9%)	0 (0.0%)	4 (23.5%)	10 (31.3%)
High school or vocational school	8 (11.9%)	0 (0.0%)	0 (0.0%)	8 (25.0%)
Bachelor’s degree or above	4 (6.0%)	0 (0.0%)	0 (0.0%)	4 (12.5%)
Impact on education or employment, n (%)	42 (62.7%)	9 (50.0%)	4 (23.5%)	29 (90.6%)
Suspend or absent from school	29 (43.3%)	9 (50.0%)	4 (23.5%)	16 (50.0%)
Unemployment	13 (19.4%)	0 (0.0%)	0 (0.0%)	13 (40.6%)
*Marital status, n (%)*				
Unmarried	63 (94.0%)	18 (100.0%)	17 (100.0%)	28 (87.5%)
Married	4 (6.0%)	0 (0.0%)	0 (0.0%)	4 (12.5%)
*Annual household income, n (%)*				
Below $4,237	14 (24.6%)	1 (5.6%)	3 (17.6%)	10 (31.3%)
$4,237—$7,062	19 (28.4%)	3 (16.7%)	7 (41.2%)	9 (28.1%)
$7,062 – $14,124	22 (32.8%)	7 (38.9%)	5 (29.4%)	10 (31.3%)
More than $14,124	12 (17.9%)	7 (38.9%)	2 (11.8%)	3 (9.4%)
Family history, n (%)	0 (0.0%)	0 (0.0%)	0 (0.0%)	0 (0.0%)

Fibrodysplasia Ossificans Progressiva, FOP; ^1^Basic medical insurance included urban employee basic medical insurance and urban and rural resident basic medical insurance.^2^Other insurance included commercial health insurance and supplementary medical insurance

Subgroup results showed the average ages of patients in the < 8, 8–15, and ≥ 16 years subgroups were 5.7 ± 1.2, 11.5 ± 1.9, and 25.5 ± 7.4 years, respectively. Only 12.5% of adult patients had a bachelor’s degree or above as their highest educational level. In patients aged < 8 and 8–15 years, 50% and 23.5% were suspended or absent from school respectively. Additionally, 40.6% of those aged ≥ 16 years were unemployed due to FOP. **(**Table 1**).**

### Diagnosis and prognosis

The average age of onset in the total FOP population was 4.9 ± 4.2 years, with a delayed duration for diagnosis of 3.1 ± 4.3 years on average. Up to 78.8% of patients would have been misdiagnosed at least once, with tumors (40.9%) being the most common misdiagnosed condition. 62.7% of patients received iatrogenic harm, with surgeries (26.9%), biopsies (25.4%), and punctures (17.9%) being the most common iatrogenic injuries experienced. 59.7% of patients had the FOP classic mutation, while 23.9% of patients had an unknown mutation type (Table 2). Only 23.9% of patients received a correct diagnosis at their initial hospital visit **(**Table S1**).**

**Table 2:** Diagnosis and prognosis of FOP patients

Variables	Total population N = 67	Age < 8 N = 18	Age 8–15 N = 17	Age ≥ 16 N = 32
*Diagnosis*				
Age of onset, years, mean (SD)	4.9 (4.2)	2.3 (1.3)	5.1 (3.7)	6.3 (4.9)
95% Confidence intervals	(3.9,6.0)	(1.6,3.0)	(3.1,7.0)	(4.5,8.1)
Median (range)	3.7 (0.1,15.2)	1.8 (0.6,4.9)	4.5 (1.1,11.5)	4.7 (0.1,15.2)
Delayed diagnosis duration, years, mean (SD)	3.1 (4.3)	0.7 (0.9)	1.1 (1.2)	5.5 (5.2)
95% Confidence intervals	(2.0,4.1)	(0.2,1.1)	(0.5,1.7)	(3.7,7.4)
Median (range)	1.1 (0,18.0)	0.4 (0,4.1)	0.7 (0,4.1)	4.1 (0,18.0)
Disease duration, years, mean (SD)	8.7 (7.9)	2.8 (1.6)	5.4 (2.8)	13.7 (8.6)
95% Confidence intervals	(6.7,10.6)	(2.0,3.5)	(4.0,6.8)	(10.6,16.8)
Median (range)	6.5 (0.6,43.8)	2.5 (0.6,6.1)	6.5 (0.7,10.7)	13.9 (0.6,43.8)
Misdiagnosis rate^1^, n (%)	52 (78.8%)	18 (100.0%)	13 (76.5%)	21 (65.6%)
Misdiagnosed as cancer^2^	27 (40.9%)	9 (50.0%)	6 (35.3%)	12 (37.5%)
Misdiagnosed as other diseases^3^	30 (45.5%)	9 (50.0%)	8 (47.1%)	13 (40.6%)
Iatrogenic harm, n (%)	42 (62.7%)	12 (66.7%)	9 (52.9%)	21 (65.6%)
Surgery	18 (26.9%)	3 (16.7%)	4 (23.5%)	11 (34.4%)
Biopsy	17 (25.4%)	6 (33.3%)	3 (17.6%)	8 (25.0%)
Puncture	12 (17.9%)	5 (27.8%)	3 (17.6%)	4 (12.5%)
Other	7 (10.4%)	2 (11.1%)	1 (5.9%)	4 (12.5%)
*Type of FOP, n (%)*				
FOP classic (R206H mutation)	40 (59.7%)	16 (88.9%)	14 (82.4%)	10 (31.3%)
FOP variant	11 (16.4%)	1 (5.6%)	2 (11.8%)	8 (25.0%)
Type not known/not sure	16 (23.9%)	1 (5.6%)	1 (5.9%)	14 (43.8%)
*Prognosis*				
Heterotopic ossification, n (%)	65 (97.0%)	18 (100.0%)	16 (94.1%)	31(96.9%)
Complications, n (%)	62 (92.5%)	14 (77.8%)	17 (100%)	31 (96.6%)
*Disability level*				
Disabled due to illness, n (%)	66 (98.5%)	17 (94.4%)	17 (100.0%)	32 (100.0%)
Disability certification^4^, n (%)	43 (64.2%)	4 (22.2%)	11 (64.7%)	28 (87.5%)
Using assistive devices, n (%)	30 (44.8%)	6 (33.3%)	7 (41.2%)	17 (53.1%)
Require care from caregivers, n (%)	64 (95.5%)	17 (94.4%)	16 (94.1%)	31 (96.9%)
Average number of caregivers, mean (SD)	1.6 (0.9)	1.4 (0.9)	1.6 (1.2)	1.7 (0.8)
95% Confidence intervals	(1.3,1.8)	(0.9,1.8)	(1.0,2.2)	(1.4,1.9)
Median (range)	1.0 (0,5)	1.0 (0,4)	1.0 (0,5)	2.0 (0,3)

Fibrodysplasia Ossificans Progressiva, FOP ^1^Data for one patient is unavailable due to a long time lapse, making it challenging to recall the specifics of the misdiagnosis.^2^Patients misdiagnosed as tumors may have been wrongly identified as conditions such as osteochondroma, multiple fibromatosis, myofibroblastic tumor, lymphatic malformation, et al. ^3^Apart from those misdiagnosed as tumors, 30 patients were also misdiagnosed with approximately 25 different conditions, including hematoma, synovitis, ankylosing spondylitis, torticollis, and others

1. all patients were physical type disability

The results of three subgroup analyses showed that the onset age of patients was early (age < 8, 2.3 ± 1.3 years; age 8–15, 5.1 ± 3.7; age ≥ 16, 6.3 ± 4.9). All subgroups showed high misdiagnosed rates and long delayed diagnosis duration among FOP patients, with patients aged ≥ 16 having the longest delayed diagnosis duration. More than 80% of patients aged < 8 and 8–15 years were the classic genetic mutation, while 43.8% of ≥ 16 years patients were unaware or not sure of their type of genetic mutation (Table 2). Subgroup results of FOP patients’ first visit department and first diagnosis department, etc., were reported in the Supplementary (Table S1).

For the prognosis, as high as 97.0% total population had heterotopic osteogenesis (HO), with the back (80.6%), neck (76.1%), and shoulders (68.7%) being the most common sites (Table 2 and Table S2). 92.5% of total patients had complications, with the highest proportion of complications being spinal deformities (80.6%). FOP patients generally exhibited higher levels of disability, with 98.5% of them being disabled due to FOP and 64.2% holding disability certifications related to physical impairments. 44.8% of patients required assistive devices for their daily activities, with walkers (17.9%) and wheelchairs (14.9%) being the most used assistive tools. Moreover, a substantial 95.5% of patients relied on caregivers for caregiving. The subgroup analysis results revealed that all subgroup patients had a high prevalence of HO and complications. Patients aged ≥ 16 years had the highest level of disability (Table 2 and Table S2).

### Healthcare resource utilization and economic burden

**Fig. 1:**
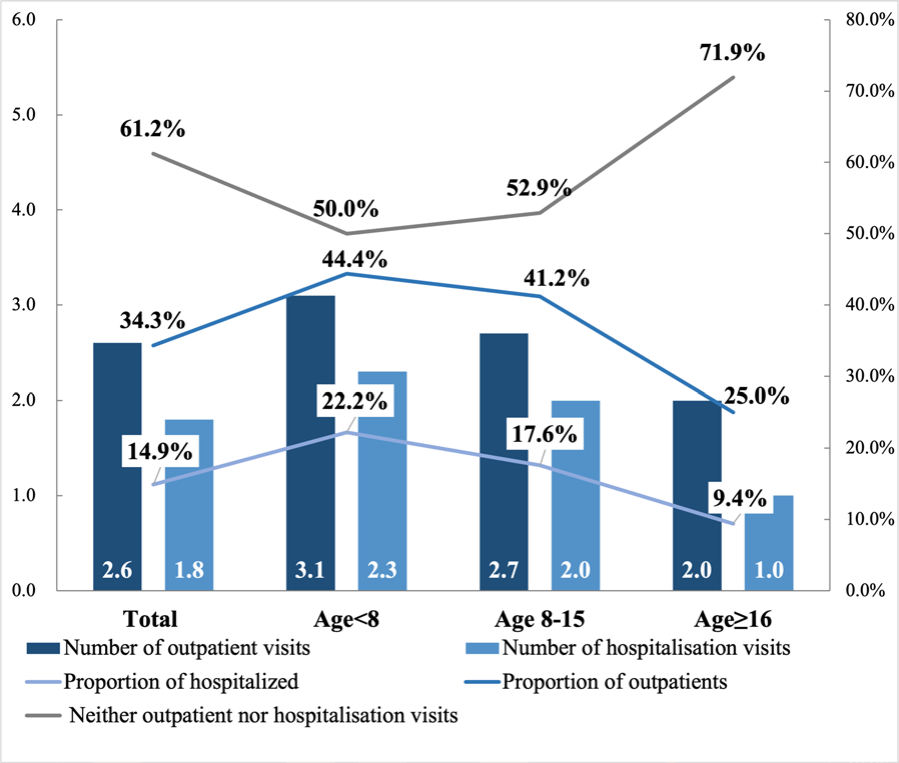
Annual healthcare resource utilization of FOP patients

**Fig. 2:**
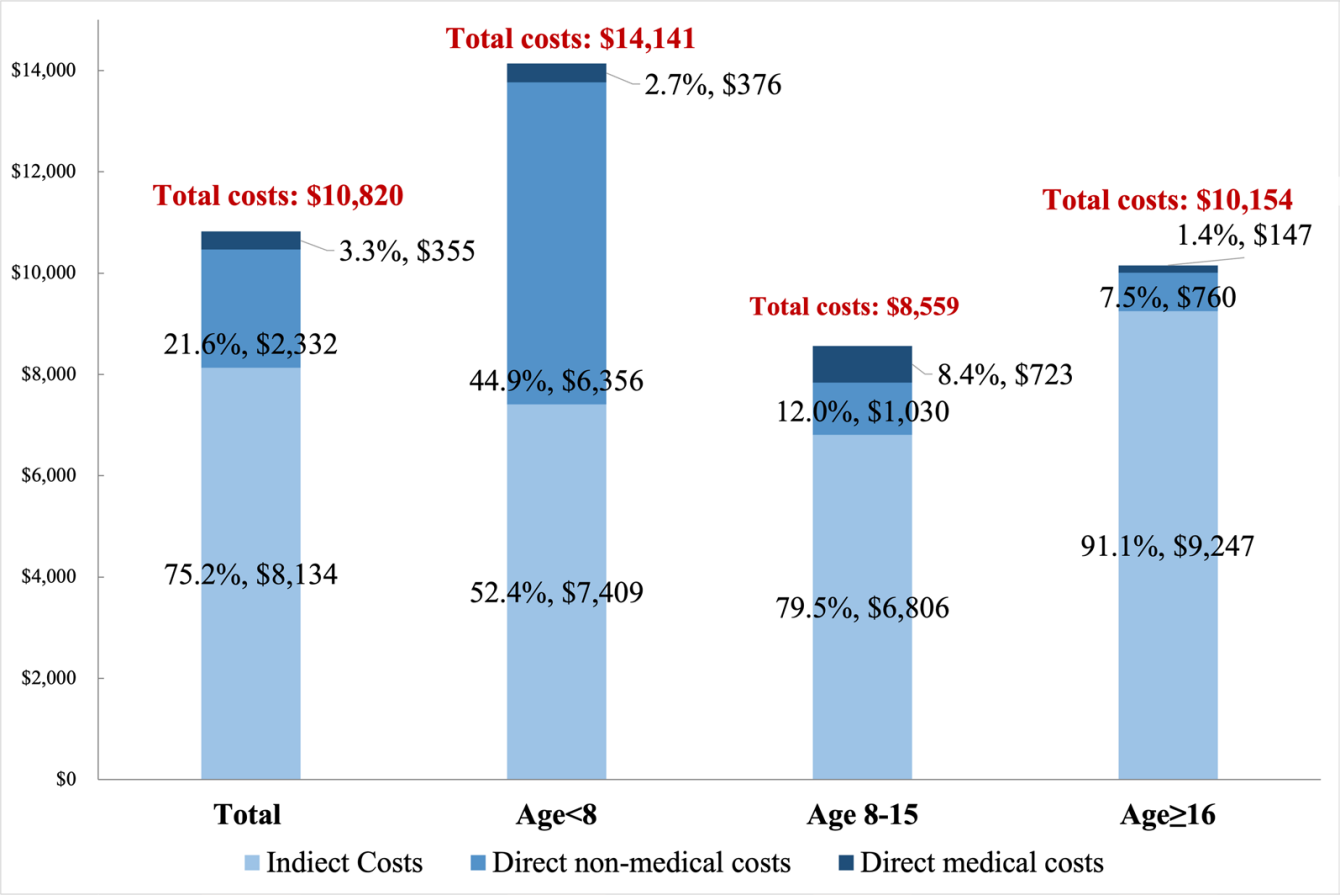
Annual costs and cost structure of FOP patients

In the past year, up to 61.2% of patients had neither outpatient nor inpatient visits due to FOP. Only 14.9% of patients had hospital admissions, with an annual average of 1.8 ± 1.6 hospitalizations per patient. 34.3% of patients had outpatient visits, with an annual average of 2.6 ± 1.9 outpatient visits per patient (Fig. 1). The average total annual cost was $10,820 ± 10,894, of which indirect and direct non-medical costs accounted for 75.2% ($8,134 ± 7,978) and 21.6% ($2,332 ± 7,374), respectively (Fig. 2). Detailed healthcare resource utilization and cost data were shown in the supplementary (Table S1).

Results from subgroup analysis indicated that outpatient and hospitalisation healthcare resource utilization was highest in the age < 8 years group, followed by the 8–15 years group and the ≥ 16 years group (Fig. 1). For patients aged < 8 years and 8–15 years, the main cost components arose from productivity loss in indirect costs incurred by caregivers totaling $7,409 ± 6,406 (52.4%) and $6,806 ± 8,367 (79.5%), respectively. However, for patients aged ≥ 16, the highest costs were associated with indirect costs incurred by the patients themselves and by the patient's caregivers, amounting to $5,132 ± 6,294 (50.5%) and $4,115 ± 5,937 (40.5%), respectively (Figs. S2, S3 and S4). Detailed cost data were shown in the supplementary (Table S2).

### Health-related quality of life

**Fig. 3:**
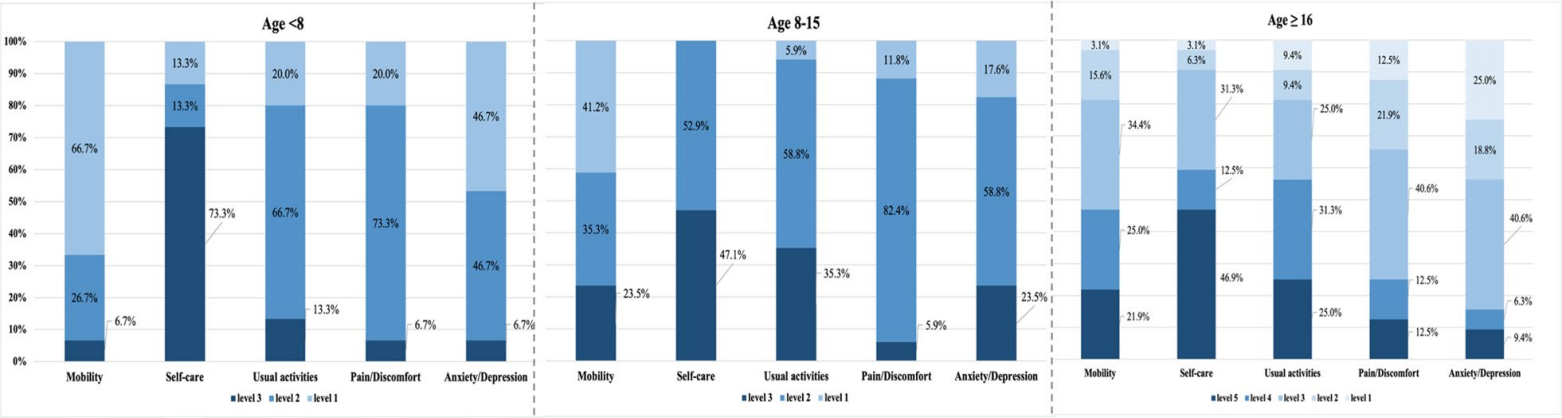
Distribution across levels of the EQ-5D dimensions of < 8, 8–15, and ≥ 16 years patients

The average health utility values of patients < 8 and 8–15 years were 0.700 ± 0.163, 0.618 ± 0.202 measured by EQ-5D-Y. The utility values of patients ≥ 16 years were 0.221 ± 0.336 measured by EQ-5D-5L, significantly lower than the younger patients (Table 3). The self-care dimension exhibited the most pronounced impairment in overall quality of life, affecting 86.7% of patients aged < 8 years, 100% of those aged 8–15 years, and 96.9% of those aged ≥ 16 years. Compared to other dimensions, it showed the highest proportion of patients with the most severe impairment across all subgroups: 73.3% (< 8 years), 47.1% (8–15 years), and 46.9% (≥ 16 years). Mobility was progressively impaired with age, with 33.3%, 58.8%, and 96.9% of patients impaired in 3 subgroups, respectively. The proportion of patients reporting damage in the usual activities dimension was 80.0%, 94.1%, and 90.6% in the 3 groups, respectively, and the proportions were similar in pain/discomfort dimension. Patients also reported high percentages of damage in the anxiety/depression dimension (53.3–82.4%) (Fig. 3).

**Table 3:** Quality of life of FOP patients

Variables	Total population^1^ N = 64	Age < 8 N = 15	Age 8–15 N = 17	Age ≥ 16 N = 32
Pediatric patients, n (%)	32 (47.8%)			
EQ-5D-Y^2^, mean (SD)	0.656 (0.187)	0.700 (0.163)	0.618 (0.202)	–
95% Confidence intervals	(0.590,0.724)	(0.609,0.790)	(0.514,0.722)	–
Median (range)	0.740 (0.193,0.910)	0.737 (0.326,0.910)	0.742 (0.193,0.838)	–
Adult patients, n (%)	32 (47.8%)			
EQ-5D-5L^3^, mean (SD)	0.221 (0.336)	–	–	0.221 (0.336)
95% Confidence intervals	(0.100,0.343)	–	–	(0.100,0.343)
Median (range)	–	–	–	0.204 (− 0.391,0.842)
≤ 4 Years patients, n (%)	3 (4.48%)	–	–	–

FOP, Fibrodysplasia Ossificans Progressiva,;^1^In the case of three patients aged below four years, quality of life conditions could not be assessed using EQ-5D.^2^The EQ-5D-Y utility values range from − 0.089 to 1. ^3^EQ-5D-5L utility values range from − 0.391 to 1

## Discussion

The current study quantified the diagnosis status, prognosis, and disease burden of FOP patients in China. We found that patients with FOP have poor diagnosis management and prognosis, heavy economic burden leading by indirect costs, and impaired health-related quality of life, which significantly affects their and caregiver’s daily life, education, and employment.

Poor disease management of FOP patients was found indicated by a high misdiagnosis rate, long diagnosis delay, and common iatrogenic injuries, which result in poor prognosis and quality of life. The duration of delay in confirmed diagnosis found in our study among patients with FOP (3.1 years) aligns with findings from earlier international studies (2.5–4.1 years) [6, 11, 12, 19]. The delay in diagnosis was shorter among patients under 15 years (0.7–1.1 years) compared to patients aged 16 years or older (4.7 years). Our misdiagnosis rate (78.8%) was lower than that reported in an earlier Chinese study (84% in 2013) and a global study (87% in 2005). [6, 20]. Consistent with findings from an international research involving 25 countries (67% in 2005), we also found a significant percentage of iatrogenic harms in China (67.2%) [6]. Based on the findings, we recommend enhancing the education and publicity of FOP among primary care physicians and establishing a robust upward referral system. And we recommend developing intelligent alert modules within electronic health record (EHR) systems to trigger FOP diagnostic prompts and recommendations for ACVR1 genetic testing based on symptom keywords such as'progressive joint stiffness'. These modules should simultaneously flag contraindicated procedures (e.g., intramuscular injections) to prevent iatrogenic harm.

Patients with FOP are not seeking health interventions actively in China. More than half of the patients did not have any outpatient visits or hospitalizations in the past year, which is lower than international research involving 15 countries’ findings. This international study indicated that 78.3% of patients consulted with general practitioners in the 12 months preceding the survey [11]. This may be due to the absence of effective drugs in this disease area. The existing medications recommended by international guidelines include corticosteroids, non-steroidal anti-inflammatory drugs (NSAIDs), aleukotriene inhibitors, mast cell stabilizers, etc. These drugs used to control symptoms of acute flare-ups in FOP, or chronic arthropathy have limited effects, but aren’t able to alter the natural history of FOP [1]. This research recommends establishing incentive policies for orphan drug research and development, such as patent term extensions and preferential tax schemes, to encourage pharmaceutical enterprises to increase their investment in the development and production of drugs for rare diseases, thereby driving innovation in breakthrough treatments for rare diseases.

The prognosis for FOP patients is unfavorable as evidenced by high disability rates, significant proportions of patients experiencing heterotopic ossification, and substantial burden of complications. A substantial majority of patients exhibit high demands for personal care tools/aids (71.3%) and care attendants (61.1%), which was in line with results from previous research [9, 10]. The poor prognosis further leads to absentiseem from school, unemployment (40.6%), and high indirect costs. The proportion of patients with a bachelor’s degree or above (12.5%) among patients aged ≥ 16 in China is considerably lower than a global study involving 15 countries (29.1%) [11]. The economic burden in patients aged < 8 and 8–15 years primarily originated from caregivers'indirect costs, while in those aged ≥ 16 years, it arose from both patients'and caregivers'indirect costs. The study recommends enhancing special education support for patients aged < 16 years and improving online education systems tailored for rare diseases such as FOP. For patients aged ≥ 16 years, employment support should be prioritized through a dedicated rare disease job platform to match patients'skills with enterprise demands, supporting flexible working arrangements such as task-based or hourly work modes.

The quality of life of FOP patients is severely damaged, with self-care being the most severely affected dimension. Two studies from Japan (n = 8) and Mexico (n = 15), utilizing the SF-36 scale, identified physical health as the most impacted dimension in patients with FOP [13, 14]. This discrepancy may be attributed to variations in scale application and the relatively small sample sizes in the two additional studies. Similar to other rare diseases, usual activities and self-care dimensions are also damaged seriously [21]. Anxiety/Depression was the relatively less damaged dimension, which was however also associated with high impairment rates. Due to the progressive nature of the disease, patients aged ≥ 16 experience the greatest impairment in the dimension of mobility compared to younger subgroups. The utility score of patients ≥ 16 years is significantly lower than that of the overall utility score (0.691) of rare disease patients measured by EQ-5D-5L [22]. In contrast, the utility scores of patients under 8 years old and those aged 8–15 years are higher than the overall utility score (0.53) of Chinese rare disease patients measured by EQ-5D-3L, which may be due to disease progression [21, 23].

The study has the following main limitations. Firstly, the study employed a retrospective approach relying on patient self-reports. It is acknowledged that patients may be susceptible to recall bias, potentially introducing a degree of deviation in the results. Secondly, as we did not collect detailed resource use and unit cost data to assess the economic burden, this might affect the result accuracy. Furthermore, the study did not evaluate the proportion of medical insurance funds and patients'out-of-pocket costs. Future research could further explore. Thirdly, there may be sample selection bias among the three subgroups, and the relatively small sample sizes in each group impose limitations on the generalizability of the results. Finally, in assessing patients'quality of life, the < 8 and 8–15 age subgroups utilized the EQ-5D-Y scale, while the ≥ 16 age subgroup used the EQ-5D-5L scale. The use of different scales for assessing quality of life may result in results that are not directly comparable. Future research could further explore the quality of life in patients across different age groups.

## Conclusions

According to patient-reported outcomes, FOP patients experience suboptimal diagnosis management, poor prognosis, substantial economic burden, and damaged quality of life. With advancing age, the disease burden exhibits a progressive trend. Efforts should be made to enhance the level of disease diagnosis, support innovative drug development and access, and establish corresponding health security measures to meet the needs of patients with FOP. Additionally, it is important to improve the educational and occupational security system for patients with FOP, enhance their social value, and facilitate their reintegration into society.

## Supplementary Information

Additional file 1.

## Data Availability

The datasets generated and/or analysed during the current study are not publicly available due to individual privacy.
