# Supplementary material for: Assessing the economic burden and health-related quality of life in chinese patients with fibrodysplasia ossificans progressiva: a questionnaire survey analysis
Source: Orphanet J Rare Dis. 2025 Aug 7;20:411. doi: 10.1186/s13023-025-03884-0 (PMC12333074; doi:10.1186/s13023-025-03884-0)
Supplement: Supplementary file 1 — Additional file 1. [file 13023_2025_3884_MOESM1_ESM.docx]

**Supplementary Table S1: Diagnosis and prognosis of FOP patients**

| **Variables** | **Total Population N=67** | **Age <8 N=18** | **Age 8-15 N=17** | **Age≥16 N=32** |
| --- | --- | --- | --- | --- |
|  |  |  |  |  |
| **First Onset Status** |  |  |  |  |
| Site of First Acute Flare-up, n (%) |  |  |  |  |
| Neck | 35 (52.2%) | 12 (66.7%) | 10 (58.8%) | 13 (40.6%) |
| Back | 18 (26.9%) | 6 (33.3%) | 4 (23.5%) | 8 (25.0%) |
| Shoulders | 9 (13.4%) | 2 (11.1%) | 4 (23.5%) | 3 (9.4%) |
| Hips | 7 (10.4%) | 1 (5.6%) | 2 (11.8%) | 4 (12.5%) |
| Elbows | 3 (4.5%) | 0 (0.0%) | 1 (5.9%) | 2 (6.3%) |
| Orther Site | 5 (7.5%) | 1 (5.6%) | 0 (0.0%) | 4 (12.5%) |
| First Acute Flare-up Symptoms, n (%) |  |  |  |  |
| Swelling | 58 (86.6%) | 18 (100.0%) | 13 (76.5%) | 27 (84.4%) |
| Restricted mobility | 19 (28.4%) | 3 (16.7%) | 6 (35.3%) | 10 (31.3%) |
| Pain | 8 (11.9%) | 1 (5.6%) | 2 (11.8%) | 5 (15.6%) |
| Fever | 5 (7.5%) | 2 (11.1%) | 0 (0.0%) | 3 (9.4%) |
| Seeking Immediate Medical Care after first flare-up, n (%) | 57 (85.1%) | 17 (94.4%) | 14 (82.4%) | 26 (81.3%) |
| Interval to Medical Care After Qnset, days, mean (SD) | 61.5 (445.9) | 1.7 (7.1) | 18.8 (51.8) | 117.8 (644.6) |
| 95% Confidence interval | [-47.2,170.2] | [-1.8,5.2] | [-7.8,45.5] | [-114.6,350.2] |
| Receiving Off-Site Medical Care, n (%) | 59 (88.1%) | 17 (94.4%) | 14 (82.4%) | 28 (87.5%) |
| **Diagnosis status of FOP patients** |  |  |  |  |
| First visit department |  |  |  |  |
| Pediatrics | 21 (31.3%) | 8 (44.4%) | 7 (41.2%) | 6 (18.8%) |
| Orthopedics | 19 (28.4%) | 2 (11.1%) | 4 (23.5%) | 13 (40.6%) |
| Surgey | 8 (11.9%) | 3 (16.7%) | 2 (11.8%) | 3 (9.4%) |
| Immunology | 6 (9.0%) | 1 (5.6%) | 2 (11.8%) | 3 (9.4%) |
| Endocrinology | 6 (9.0%) | 1 (5.6%) | 2 (11.8%) | 3 (9.4%) |
| Other | 8 (11.9%) | 3 (16.7%) | 2 (11.8%) | 3 (9.4%) |
| First diagnosis department |  |  |  |  |
| Endocrinology | 20 (29.9%) | 2 (11.1%) | 6 (35.3%) | 12 (37.5%) |
| Orthopedics | 13 (19.4%) | 2 (11.1%) | 3 (17.6%) | 8 (25.0%) |
| Immunology | 12 (17.9%) | 5 (27.8%) | 3 (17.6%) | 4 (12.5%) |
| Pediatrics | 8 (11.9%) | 3 (16.7%) | 2 (11.8%) | 3 (9.4%) |
| Rare disease center | 6 (9.0%) | 3 (16.7%) | 1 (5.9%) | 2 (6.3%) |
| Other | 8 (11.9%) | 3 (16.7%) | 2 (11.8%) | 3 (9.4%) |
| Patients Diagnosed at Initial Visit Hospital, n (%) | 16 (23.9%) | 2 (11.1%) | 5 (29.4%) | 9 (28.1%) |

**Supplementary Table S1: Diagnosis and prognosis of FOP patients (continued)**

| **Variables** | **Total Population N=67** | **Age <8 N=18** | **Age 8-15 N=17** | **Age≥16 N=32** |
| --- | --- | --- | --- | --- |
|  |  |  |  |  |
| **Location of Heterotopic Ossification, n (%)** | 65 (97.0%) | 18 (100.0%) | 16 (94.1%) | 31(96.9%) |
| Back | 54 (80.6%) | 15 (83.3%) | 15 (88.2%) | 24 (75.0%) |
| Neck | 51 (76.1%) | 15 (83.3%) | 12 (70.6%) | 24 (75.0%) |
| Shoulder | 46 (68.7%) | 11 (61.1%) | 15 (88.2%) | 20 (62.5%) |
| Elbow | 36 (53.7%) | 5 (27.8%) | 9 (52.9%) | 22 (68.8%) |
| Jaw | 29 (43.3%) | 3 (16.7%) | 9 (52.9%) | 17 (53.1%) |
| Chest | 28 (41.8%) | 8 (44.4%) | 9 (52.9%) | 11 (34.4%) |
| Knee | 25 (37.3%) | 3 (16.7%) | 6 (35.3%) | 16 (50.0%) |
| Hip | 24 (35.8%) | 3 (16.7%) | 8 (47.1%) | 13 (40.6%) |
| Abdomen | 22 (32.8%) | 8 (44.4%) | 3 (17.6%) | 11 (34.4%) |
| Wrist | 18 (26.9%) | 3 (16.7%) | 6 (35.3%) | 9 (28.1%) |
| Ankle | 14 (20.9%) | 1 (5.6%) | 4 (23.5%) | 9 (28.1%) |
| Other^2^ | 15 (22.4%) | 5 (27.8%) | 1 (5.9%) | 9 (28.1%) |
| Complications, n (%) | 62 (92.5%) | 14 (77.8%) | 17 (100%) | 31 (96.6%) |
| Spinal Deformities | 54 (80.6%) | 11 (61.1%) | 15 (88.2%) | 28 (87.5%) |
| Temporomandibular Joint Stiffness | 31 (46.3%) | 3 (16.7%) | 9 (52.9%) | 19 (59.4%) |
| Hearing Impairment | 27 (40.3%) | 2 (11.1%) | 8 (47.1%) | 17 (53.1%) |
| Thoracic Insufficiency Syndrome | 11 (16.4%) | 1 (5.6%) | 4 (23.5%) | 6 (18.8%) |
| Pressure Ulcer | 8 (11.9%) | 0 (0.0%) | 1 (5.9%) | 7 (21.9%) |
| Pneumonia | 7 (10.4%) | 3 (16.7%) | 1 (5.9%) | 3 (9.4%) |
| Other^3^ | 3 (4.5%) | 2 (11.1%) | 0 (0.0%) | 1 (3.1%) |
| Disability Level |  |  |  |  |
| Disability Certificate Level, n (%) |  |  |  |  |
| Level 1 | 11 (16.4%) | 0 (0.0%) | 1 (5.9%) | 10 (31.3%) |
| Level 2 | 23 (34.3%) | 2 (11.1%) | 6 (35.3%) | 15 (46.9%) |
| Level 3 | 7 (10.4%) | 1 (5.6%) | 3 (17.6%) | 3 (9.4%) |
| Level 4 | 2 (3.0%) | 1 (5.6%) | 1 (5.9%) | 0 (0.0%) |
| Using Assistive Devices Patients, n (%) | 30 (44.8%) | 6 (33.3%) | 7 (41.2%) | 17 (53.1%) |
| Walker (such as crutches, etc.) | 12 (17.9%) | 0 (0.0%) | 1 (5.9%) | 11 (34.4%) |
| Wheelchair | 10 (14.9%) | 2 (11.1%) | 4 (23.5%) | 4 (12.5%) |
| Hearing Aid | 4 (6.0%) | 0 (0.0%) | 1 (5.9%) | 3 (9.4%) |
| Breathing Trainer | 1 (1.5%) | 1 (5.6%) | 0 (0.0%) | 0 (0.0%) |
| Oxygen Concentrator | 1 (1.5%) | 0 (0.0%) | 1 (5.9%) | 2 (6.3%) |
| Home or Vehicle Modifications | 2 (3.0%) | 0 (0.0%) | 0 (0.0%) | 2 (6.3%) |
| Orther^2^ | 6 (9.0%) | 3 (16.7%) | 2 (11.8%) | 1 (3.1%) |

**Supplementary Table S2: Healthcare resource utilization and costs of FOP patients**

| **Variables** | **Total Population N=67** | **Age <8 N=18** | **Age 8-15 N=17** | **Age≥16 N=32** |
| --- | --- | --- | --- | --- |
| **Healthcare resource utilization over the last year** |  |  |  |  |
| **Patients without hospitalization and outpatient visits, n (%)** | 41 (61.2%) | 9 (50.0%) | 9 (52.9%) | 23 (71.9%) |
| **Hospitalized Patients, n (%)** | 10 (14.9%) | 4 (22.2%) | 3 (17.6%) | 3 (9.4%) |
| Number of hospitalizations among hospitalized patients, mean (SD) | 1.8 (1.6) | 2.3 (2.5) | 2.0 (1.0) | 1.0 (0.0) |
| 95% Confidence interval | (0.6,3.0) | (-1.7,6.2) | (-0.48,4.5) | (1,1) |
| Median (range) | 1.0 (1,6) | 1.0 (1,6) | 2.0 (1,3) | 1.0 (1,1) |
| **Patients with Outpatient Visits, n (%)** | 23 (34.3%) | 8 (44.4%) | 7 (41.2%) | 8 (25.0%) |
| Number of Outpatient Visits, mean (SD) | 2.6 (1.9) | 3.1 (2.2) | 2.7 (2.1) | 2.0 (1.4) |
| 95% Confidence interval | (1.8,3.4) | (1.3,4.9) | (0.8,4.6) | (0.8,3.2) |
| Median (range) | 2.0 (1,7) | 3.0 (1,6) | 2.0 (1,7) | 1.5 (1,5) |
| **Total Annual Costs, $,mean (SD)** | 10820(10894) | 14141(15071) | 8559(8970) | 10154(8771) |
| 95% Confidence interval | (8163,13478) | (6646,21636) | (3947,13171) | (6992,13316) |
| Median (range) | 12621 (0,50756) | 12840 (56,50756) | 3811 (0,25689) | 12621 (0,27492) |
| **Direct Medical Costs, $,mean (SD)** | 355 (791) | 376 (595) | 723 (1308) | 147 (370) |
| 95% Confidence interval | (162,548) | (80,672) | (51,1396) | (14,281) |
| Median (range) | 300 (0,3967) | 42 (0,2090) | 0 (0,3967) | 0 (0,1624) |
| **Hospitalisation, mean (SD)** | 223 (704) | 200 (421) | 557 (1266) | 57 (200) |
| 95% Confidence interval | (51,394) | (-9,410) | (-93,1208) | (-15,130) |
| Median (range) | 0 (0,3967) | 0 (0,1412) | 0 (0,3967) | 0 (0,993) |
| **Outpatient, mean (SD)** | 132 (315) | 176 (273) | 166 (390) | 90 (297) |
| 95% Confidence interval | (55,209) | (40,312) | (-34,366) | (-17,197) |

**Supplementary Table S2: Healthcare resource utilization and costs of FOP patients (continued)**

| **Variables** | **Total Population N=67** | **Age <8 N=18** | **Age 8-15 N=17** | **Age≥16 N=32** |
| --- | --- | --- | --- | --- |
| Median (range) | 0 (0,1624) | 0 (0,862) | 0 (0,1483) | 0 (0,1624) |
| **Direct Non-Medical Costs, $,mean (SD)** | 2332 (7374) | 6356 (13512) | 1030 (1324) | 760 (1366) |
| 95% Confidence interval | (533,4131) | (-364,13075) | (349,1711) | (268,1252) |
| Median (range) | 282 (0,42373) | 685 (0,42373) | 446 (0,4668) | 174 (0,5791) |
| **Transports, mean (SD)** | 197 (308) | 370 (400) | 180 (290) | 109 (213) |
| 95% Confidence interval | (122,272) | (170,569) | (31,329) | (32,186) |
| Median (range) | 25 (0,1412) | 353 (0,1412) | 25 (0,1130) | 0 (0,706) |
| **Accommodation, mean (SD)** | 218 (474) | 445 (773) | 182 (345) | 110 (217) |
| 95% Confidence interval | (103,334) | (60,830) | (5,359) | (32,188) |
| Median (range) | 14 (0,2825) | 177 (0,2825) | 14 (0,1412) | 0 (0,706) |
| **Nursing, mean (SD)** | 1442 (6611) | 5148 (12221) | 133 (548) | 53 (300) |
| 95% Confidence interval | (-171,3054) | (-930,11225) | (-149,415) | (-55,161) |
| Median (range) | 0 (0,40678) | 0 (0,40678) | 0 (0,2260) | 0 (0,1695) |
| **Nutrition, mean (SD)** | 306 (953) | 367 (1323) | 322 (725) | 263 (836) |
| 95% Confidence interval | (73,538) | (-291,1025) | (-50,695) | (-39,564) |
| Median (range) | 0 (0,5650) | 0 (0,5650) | 0 (0,2373) | 0 (0,4520) |
| **Assistive Device, mean (SD)** | 168 (429) | 27(68) | 212(495) | 225(497) |
| 95% Confidence interval | (64,273) | (-7,60) | (-42,467) | (46,404) |
| Median (range) | 0 (0,2119) | 0 (0,282) | 0 (0,1603) | 0 (0,2119) |
| **Indiect Costs, $,mean (SD)** | 8134(7978) | 7409(6406) | 6806(8367) | 9247(8622) |
| 95% Confidence interval | (6188,10080) | (4224,10595) | (2504,11107) | (6138,12355) |

**Supplementary Table S2: Healthcare resource utilization and costs of FOP patients (continued)**

| **Variables** | **Total Population N=67** | **Age <8 N=18** | **Age 8-15 N=17** | **Age≥16 N=32** |
| --- | --- | --- | --- | --- |
| Median (range) | 12621 (0, 26645) | 12621 (0,14023) | 1402 (0,25242) | 12621 (0, 26645) |
| **Patients’ indirect costs, mean (SD)** | 2451(5028) | 0 (0) | 0 (0) | 5132(6294) |
| 95% Confidence interval | (1225,3677) | (0,0) | (0,0) | (2862,7401) |
| Median (range) | 0 (0,12621) | 0 (0,0) | 0 (0,0) | 0 (0,12621) |
| Unemployment costs, mean (SD) | 2449(5029) | 0 (0) | 0 (0) | 5127(6298) |
| 95% Confidence interval | (1222,3675) | (0,0) | (0,0) | (2857,7398) |
| Median (range) | 0 (0,12621) | (0,0) | (0,0) | 0 (0,12621) |
| Absenteeism costs, mean (SD) | 2(17) | 0 (0) | 0 (0) | 4(25) |
| 95% Confidence interval | (-2,6) | (0,0) | (0,0) | (-5,13) |
| Median (range) | 0 (0,140) | 0 (0,0) | 0 (0,0) | 0 (0,140) |
| **Caregivers’ costs, mean (SD)** | 5683(6814) | 7409(6406) | 6806(8367) | 4115(5937) |
| 95% Confidence interval | (4021,7345) | (4224,10595) | (2504,11107) | (1974,6256) |
| Median (range) | 982 (0,25242) | 12621 (0,14023) | 1402 (0,25242) | 0 (0,14023) |
| Unemployment Costs, mean (SD) | 5275(6646) | 7012(6453) | 5939(7879) | 3944(5944) |
| 95% Confidence interval | (3654,6896) | (3803,10221) | (1888,9990) | (1801,6087) |
| Median (range) | 0 (0,25242) | 12621 (0,12621) | 0 (0,25242) | 0 (0,12621) |
| Absenteeism Costs, mean (SD) | 408(792) | 397(540) | 866(1266) | 171(418) |
| 95% Confidence interval | (215,601) | (129,666) | (215,1517) | (20,322) |
| Median (range) | 0 (0,4207) | 0 (0,1402) | 0 (0,4207) | 0 (0,1402) |


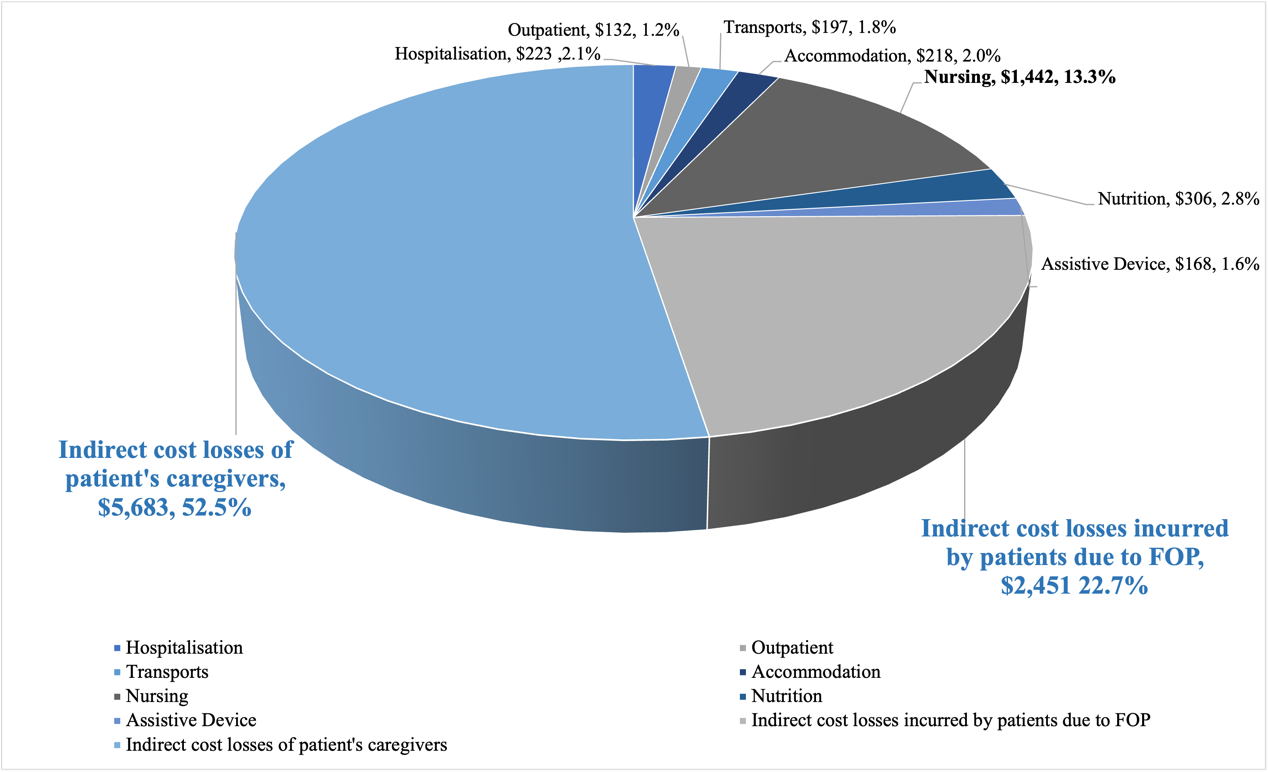


**Supplementary Figure S1: Annual cost structure of the FOP total population**

**
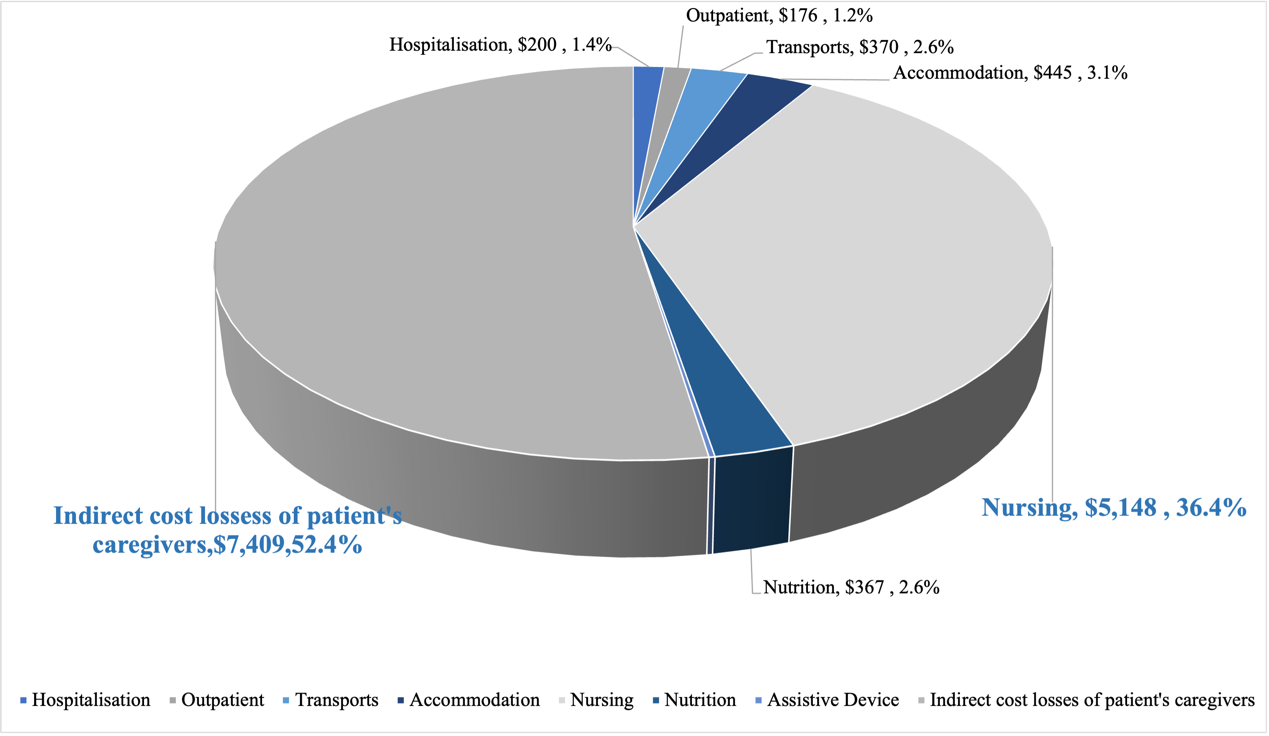
**

**Supplementary Figure S2: Annual cost structure of the < 8-year-old FOP population**

**
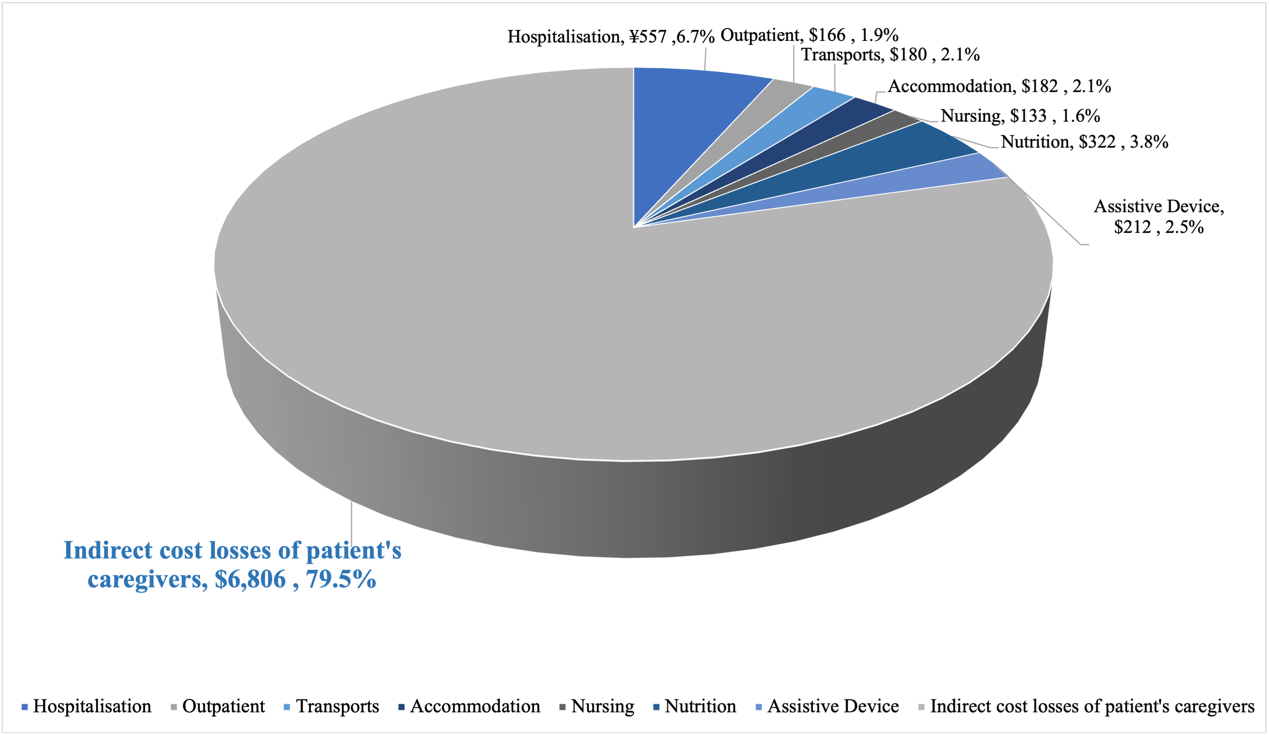
**

**Supplementary Figure S3: Annual cost structure of the 8–15-year-old FOP population**

**
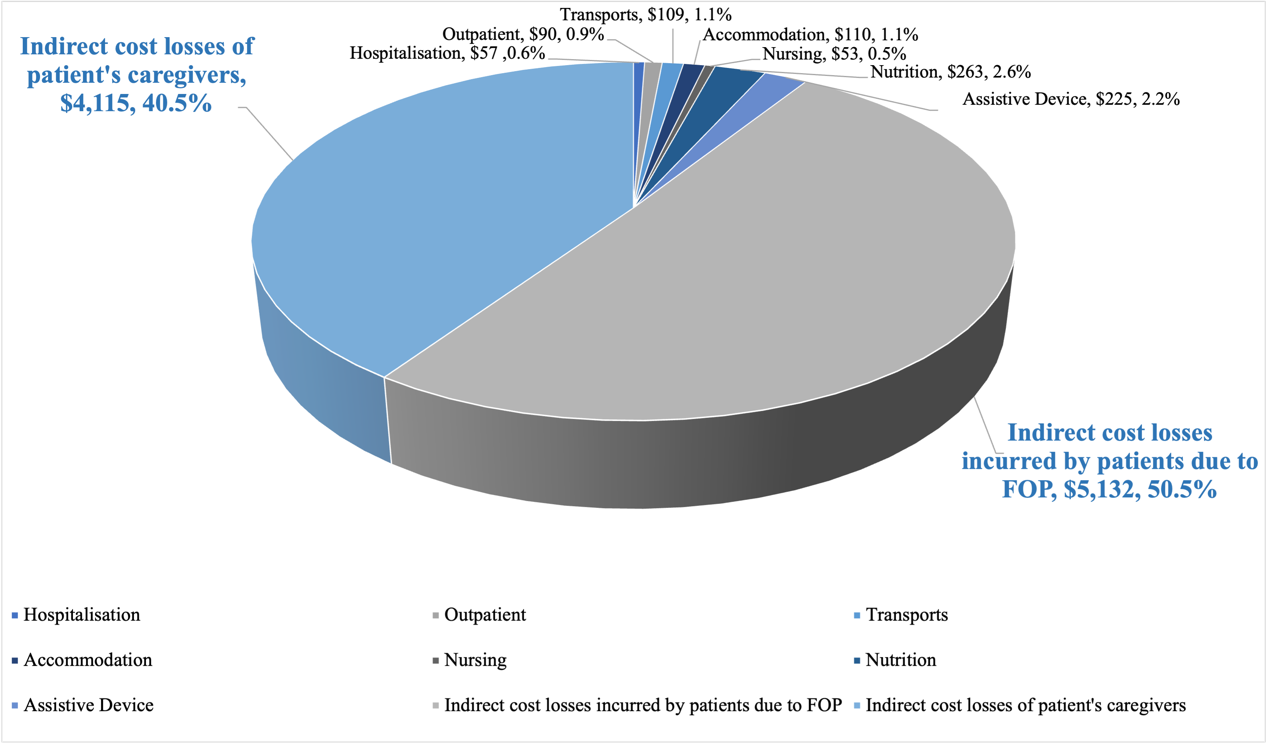
**

**Supplementary Figure S4: Annual cost structure of the ≥ 16-year-old FOP population**
